# The 10^th^ Congress of the European Academy of Neurology – 29 June – 2 July 2024

**DOI:** 10.25122/jml-2023-1026

**Published:** 2023-08

**Authors:** European Academy of Neurology

**Figure 1:**
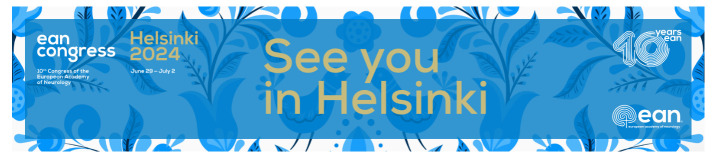
Announcement for the 10^th^ EAN Congress

The European Academy of Neurology (EAN) will open the doors to its annual congress for the tenth time in 2024, from 29 June to 2 July, marking a decade of the ‘Home of Neurology’ (Figure 1). Aside from a few exceptions during the COVID-19 lockdown years, Europe’s largest professional neurological organization has rotated its annual congress to locations across Europe since the first event in Berlin, in 2015. For its tenth anniversary, the congress will set up a temporary home of neurology in Helsinki, at Finland’s largest convention center, the Messukeskus. For the upcoming event, thousands of neurologists and other professionals will travel to Helsinki, but the congress will reach an audience far beyond those who make the journey. As has been the case for most organizers of annual congresses, the EAN faced unique challenges during the years when large gatherings were not possible, leading to innovations that have lasted beyond the pandemic. Drawing from the valuable experience of hosting entirely virtual congresses in 2020 (at very short notice) and 2021, the EAN has held hybrid events since 2022. This approach has allowed almost the entire scientific and educational program (with the obvious exception of certain hands-on sessions) to be made available online in real-time – and to watch back on demand – both for ‘virtual only’ participants and for onsite attendees. This full broadcasting service will naturally continue for the 10th edition of the EAN Congress, extending the opportunity to engage those who would otherwise not be able to benefit from the EAN’s top-quality program.

The Overarching Theme of the EAN Congress 2024 will be ‘Neuromodulation: advances and opportunities in neurological diseases’. As always, the central theme will be a common factor among all the talks provided by invited lecturers across many types of sessions, symposia, workshops, and courses. The program is designed to cater to every category of participant from trainees to specialists, for both clinicians and scientists. In addition, the EAN Programme Committee has also put together a selection of sessions with a tighter focus on this theme, in which leading specialists will tackle key topics in invasive and non-invasive neuromodulation in movement and cognitive disorders, chronic pain, and refractory epilepsy.

The EAN Congress 2024 will address the current state and future directions of neuromodulation focusing on techniques available as diagnostic tools or therapeutic interventions; how to precisely target specific cortical regions, deep brain structures, and head nerves for therapeutic purposes; and how to advance neuromodulation into new areas of neurology such as chronic minimally conscious state or limb prosthesis.

The EAN Programme Committee and Teaching Course Subcommittee, together with the Local Organising Committee, have already put in a great deal of work assembling the basis for the forthcoming congress. However, no medical congress would be complete without the contributions of its own community. Abstract submission will run from late November to January, with a ‘late-breaking abstracts’ window opening between April and June. We aim to achieve similar or greater figures than last year’s 2,318 submissions (a new record for an in-person EAN congress). With numerous presentation types, including oral presentations, ePresentations, ePosters, and virtual ePosters, there is a great range of ways to get involved in the congress and share your work with our engaged and enthusiastic international audiences.

For potential participants interested in keeping up to date with announcements regarding abstract submission, registration, and program details for the EAN Congress 2024, there is no better way to stay informed than to become an EAN member. With special membership packages for students (free of charge), residents, research fellows, non-neurologists, and neurologists working in non-EAN member countries – as well as regular ‘full’ membership and associate membership (automatically bestowed upon members of EAN’s 47 national member societies) – there are options to suit everyone. Most importantly, EAN membership offers many benefits such as free virtual congress participation for many membership categories, reduced registration fees for onsite attendance, and long-term access to online content for all, available at ean.org/join.

**Figure 2:**
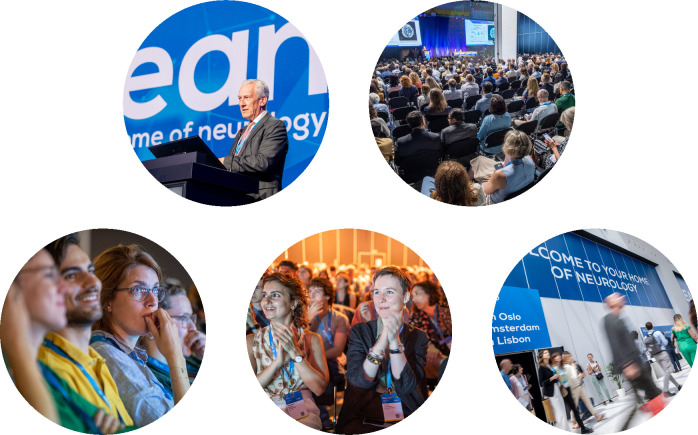
Insights from the 9^th^ Congress of the European Academy of Neurology (EAN)

**The 9^th^ Congress of the European Academy of Neurology (EAN)** (Figure 2) took place this year in Budapest, Hungary, spanning from July 1st to 4^th^. This event seamlessly integrated scientific endeavors with industry insights, in a dynamic and intellectually enriching forum, achieving resounding success in advancing neurology. Structured in a hybrid format, the EAN Congress welcomed nearly 6,000 on-site attendees and overall 7,722 participants from 116 countries. The scientific event featured a robust program of 269 sessions, including oral presentations, ePresentations, ePosters, and virtual ePosters, with 460 invited lectures and 1,912 abstracts presented across the spectrum of neurology's subspecialties. The overarching theme of the congress, ‘Neurology Beyond Big Data’ guided the exploration of neurology through recent advances, opportunities, and challenges in navigating the ever-increasing amount of information. With the advancement of technology, new avenues arise for collecting and analyzing data and promoting deeper insight into the workings of the human brain. Big data offers an amazing opportunity to improve technologies and treatments and support public health interventions.

The European Academy of Neurology has made remarkable strides over the years, fostering a culture of excellence within the realm of neurology. Its endeavors have transcended geographical boundaries, effectively bridging scientific and educational pursuits across nations while playing a pivotal role in supporting patients and their caregivers. Serving as a dynamic nexus, the EAN represents a hub for professionals across all age groups, united by a shared passion for neurology. This vibrant community not only provides a platform for career development but also contributes significantly to the deeper comprehension of neurological sciences. Regardless of your specific interest in the field of neurology, we invite you to join us in Helsinki or online in 2024 and we look forward to extending a warm welcome to what will surely be an excellent congress.

For more information about the EAN, visit ean.org. For congress information, visit ean.org/congress2024.

